# Alpinetin suppresses CYP3A4, 2C9, and 2E1 activity *in vitro*

**DOI:** 10.1080/13880209.2022.2071450

**Published:** 2022-05-28

**Authors:** Hongming Song, Chuankui Wei, Wu Yang, Zhaohe Niu, Mingkai Gong, Haiyan Hu, Haibo Wang

**Affiliations:** a Breast Disease Center, The Affiliated Hospital of Qingdao University, Qingdao, People’s Republic of China; b Department of General Surgery, The Second Affiliated Hospital of Shandong First Medical University, Taian, People’s Republic of China; c Department of International Medicine, The Affiliated Hospital of Qingdao University, Qingdao, People’s Republic of China

**Keywords:** Cytochrome P450 enzymes, non-competitive inhibition, competitive inhibition

## Abstract

**Context:**

Alpinetin, the major active constitutes of *Alpinia katsumata* Hayata (Zingiberaceae), has been demonstrated to possess the activity of anti-breast cancer. Cytochrome P450 enzymes (CYP450s) plays vital roles in the biotransformation of various drugs.

**Objective:**

To assess the effect of alpinetin on the activity of CYP450s and estimate the inhibition characteristics.

**Materials and methods:**

The activity of CYP450s was evaluated in pooled human liver microsomes with corresponding substrates and marker reactions. The effect of alpinetin was compared with blank control (negative control) and corresponding inhibitors (positive control). The dose-dependent and time-dependent experiments were conducted in the presence of 0, 2.5, 5, 10, 25, 50, and 100 μM alpinetin and incubated for 0, 5, 10, 15, and 30 min.

**Results:**

Alpinetin suppressed CYP3A4, 2C9, and 2E1 activity. All the inhibitions were significantly influenced by alpinetin contration with the IC_50_ values of 8.23 μM (CYP3A4), 12.64 μM (CYP2C9), and 10.97 μM (CYP2E1), respectively. The inhibition of CYP3A4 was fitted with the non-competitive model with a *Ki* value of 4.09 μM and was time-dependent with *KI* and *Kinact* values of 4.67 min and 0.041 μM^−1^, respectively. While CYP2C9 and 2E1 were inhibited by alpinetin competitively with *Ki* values of 6.42 (CYP2C9) and 5.40 μM (CYP2E1), respectively, in a time-independent manner.

**Discussion and conclusion:**

The *in vitro* inhibitory effect of alpineticn on CYP3A, 2C9, and 2E1 implied the potential interaction of alpinetin or its origin herbs with the drugs metabolised by those CYP450s, which needs further *in vivo* validation.

## Introduction

Breast cancer is one of the leading causes of cancer-related death in women. Although the clinical management of breast cancer has been greatly improved, metastasis and recurrency still frequently occur (Scully et al. 2012). Chemotherapy and radiotherapy gradually account for a critical position in the therapeutic strategies of breast cancer (Zhang and Li 2018). However, both these two treatments are not able to distinguish cancer cells (Staff et al. 2017). As expected, Chinese traditional medicine has been widely accepted in the therapy of various human diseases, which could avoid the side effects on normal cells. *Alpinia katsumata* Hayata (Zingiberaceae) seeds have been commonly used in the treatment of inflammation and gastric disorders (Lee et al. 2003), but its extractions have distinguished pharmacological activities (Wang et al. 2008). For example, (*E*)-methyl-cinnamate, an extraction of *A. katsumata*, were revealed to suppress pre-osteoblasts growth, migration, and differentiation (Park et al. 2020). Alpinetin, a major active ingredient of *A. katsumata*, has been reported to possess a significant antitumor activity in various human cancers, including breast cancer (Wang et al. 2016; Wu et al. 2016; Zhao et al. 2018; Hou et al. 2021; Zhang et al. 2021). It was reported that alpinetin showed an inhibitory effect on the progression of breast cancer via the ROS/NF-κB/HIF-1α axis (Zhang, Guo, et al. 2020). The interaction between active ingredients of different herbs would induce therapy failure or even drug toxicity.

As the prescription is always a mixture that includes various herbs with similar or complementary indications in traditional Chinese medicine, it makes the drug–drug interaction easier during the combination of various herbs. Cytochrome P450 enzymes (CYP450s) are a superfamily that partakes in the biotransformation of various xenobiotics, such as drugs and toxins (Manikandan and Nagini 2018). CYP450s is also a vital factor mediating the interaction between co-administrated drugs or compounds. Therefore, the influence of herb major active constituent on the activity of CYP450s is a critical basis for the clinical application of the original herbs and drugs, especially for the co-administration of different drugs (Lynch and Price 2007; Hakkola et al. 2020). Due to the miscellaneous pharmacological effects of alpinetin, especially for its anticancer activity, it is of great possibility that alpinetin would be co-administrated with other anticancer herbs. Hence, disclosing the effect of alpinetin on the activity of CYP450s would help understand the potential of alpinetin and its origin herb *A. katsumata* in inducing adverse drug-drug interaction.

Here, the interaction of alpinetin with eight major CYP450s isoenzymes was investigated in human liver microsomes, in order to guide the clinical co-administrated prescription of alpinetin and its origin herbs.

## Materials and methods

### Human liver microsomes assay

**Figure 1.**
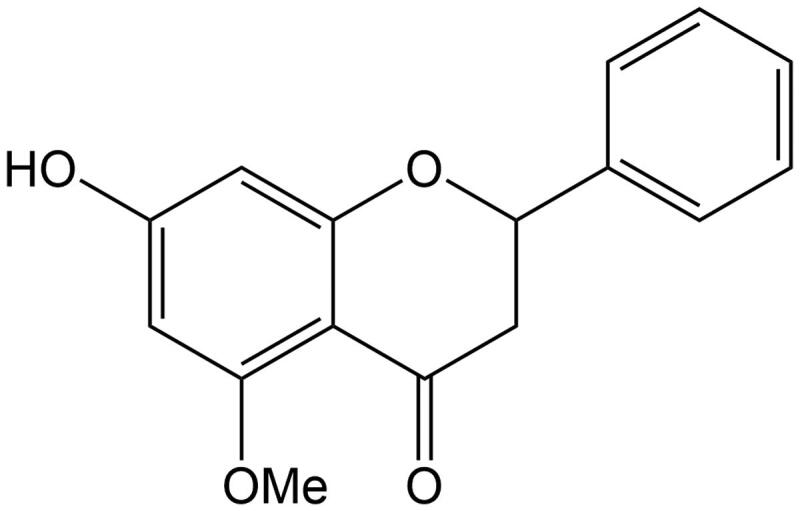
The chemical structure of alpinetin.

The pooled human liver microsomes (HLMs) were obtained from BD Biosciences Discovery Labware. The HLMs were incubated with specific substrates of corresponding CYP450 isoenzymes under the reaction conditions in Table 1 according to previous reports (Dong et al. 2018; Zhang et al. 2019; Zhang, Feng, et al. 2020). The incubation system also included alpinetin (Figure 1, 99%, Chengdu Munster Biothechnology Co, China, or specific inhibitors) and an β-NADPH generating system as previously reported in the potassium phosphate buffer with a final volume of 200 μL. The β-NADPH generating system contained NADP^+^, glucose-6-phosphate, glucose-6-phosphate dehydrogenase, and MgCl_2_, which was used to initiate the reaction after a pre-incubation of 3 min. At the end of the reactions, the acetonitrile or trichloroacetic acid (for the evaluation of CYP2A6) was added for the termination of the reactions. All reagents were of at least analytical reagent grade obtained from Sigma Aldrich (USA). All incubations were performed in triplicate and the concentrations of metabolites were analysed with HPLC.

**Table 1.** The reaction conditions of corresponding CYP450 isoforms.

CYPs	Marker reactions	Substrate concentration (μM)	Inhibitors	Protein concentration (mg/mL)	Incubation time (min)	Estimated *K*_m_ (μM)	References
1A2	Phenacetin *O*-deethylation	40	Furafylline	0.2	30	48	Dong et al. (2018), Zhang et al. (2019), Zhang, Feng et al. (2020)
2A6	Coumarin 7-hydroxylation	1.0	Tranylcypromine	0.1	10	1.5	Dong et al. (2018), Zhang et al. (2019), Zhang, Feng, et al. (2020)
3A4	Testosterone 6β-hydroxylation	50	Ketoconazole	0.5	10	53	Dong et al. (2018), Zhang et al. (2019), Zhang, Feng et al. (2020)
2C8	Paclitaxel 6α-hydroxylation	10	Montelukast	0.5	30	16	Dong et al. (2018), Zhang et al. (2019), Zhang, Feng, et al. (2020)
2C9	Diclofenac 4′-hydroxylation	10	Sulphaphenazole	0.3	10	13	Dong et al. (2018), Zhang et al. (2019), Zhang, Feng, et al. (2020)
2C19	S-Mephenytoin 4-hydroxylation	100	Tranylcypromine	0.2	40	105	Dong et al. (2018), Zhang et al. (2019), Zhang, Feng et al. (2020)
2D6	Dextromethorphan *O*-demethylation	25	Quindine	0.25	20	4.8	Dong et al. (2018), Zhang et al. (2019), Zhang, Feng, et al. (2020)
2E1	Chlorzoxazone 6-hydroxylation	120	Clomethiazole	0.4	30	126	Dong et al. (2018), Zhang et al. (2019), Zhang, Feng, et al. (2020)

### Inhibition kinetic study

The effect of alpinetin on the activity of CYP1A2, 2A6, 3A4, 2C8, 2C9, 2C19, 2D6, and 2E1 was first evaluated with a concentration of 100 μM. The isoenzymes that were inhibited by alpinetin were further incubated with 0, 2.5, 5, 10, 25, 50, and 100 μM alpinetin to obtain the values of IC_50_.

Meanwhile, the kinetic studies were conducted in the presence of various substrate concentrations to obtain the values of *Ki*. The inhibition model was evaluated according to the following equations: *v* = (*V*max*S*)/[*Km*(1 + *I*/*Ki*) + S] for the competitive inhibition and v = (*V*max*S*)/[(*Km* + S(1 + *I*/*KiKI*)] for the non-competitive inhibition. The *I* is the concentration of the compound, *Ki* is the inhibition constant, *S* is the concentration of the substrate, and *Km* is the substrate concentration at half the maximum velocity (*V*max) of the reaction.

### Time-dependent evaluation

The time-dependent inhibition assay was conducted with an incubation time of 0, 5, 10, 15, and 30 min after a 30 min pre-incubation with 1 mg/mL HLMs in the presence of NADPH-generating system at 37 °C. The concentration of corresponding substrates was close to the value of *Km*, while a higher concentration of approximate to 4-fold *Km* was used in the evaluation of the values of *KI* and *Kinact*.

### Statistical analysis

All data were presented as mean ± SD and analysed by the Student’s *t*-test or one-way ANOVA followed by the Turkey *post hoc* test with SPSS 20.0.

## Results

### Effect of alpinetin on the activity of CYP450s

**Figure 2.**
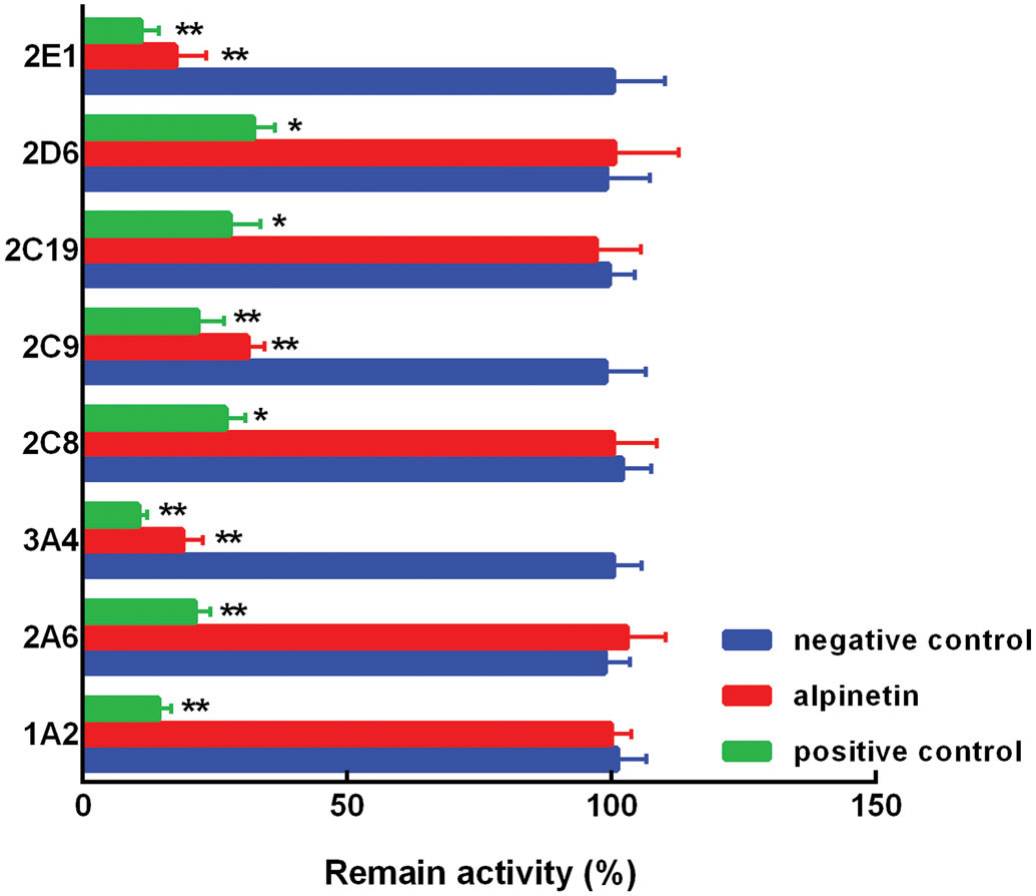
The activity of CYP1A2, 2A6, 3A4, 2C8, 2C9, 2C19, 2D6, and 2E1 in the presence of alpinetin or specific inhibitors. Negative control: without any treatments, alpinetin: with 100 μM alpinetin, positive control: with specific inhibitors. *p < 0.05, **p < 0.01 relative to the negative control.

**Figure 3.**
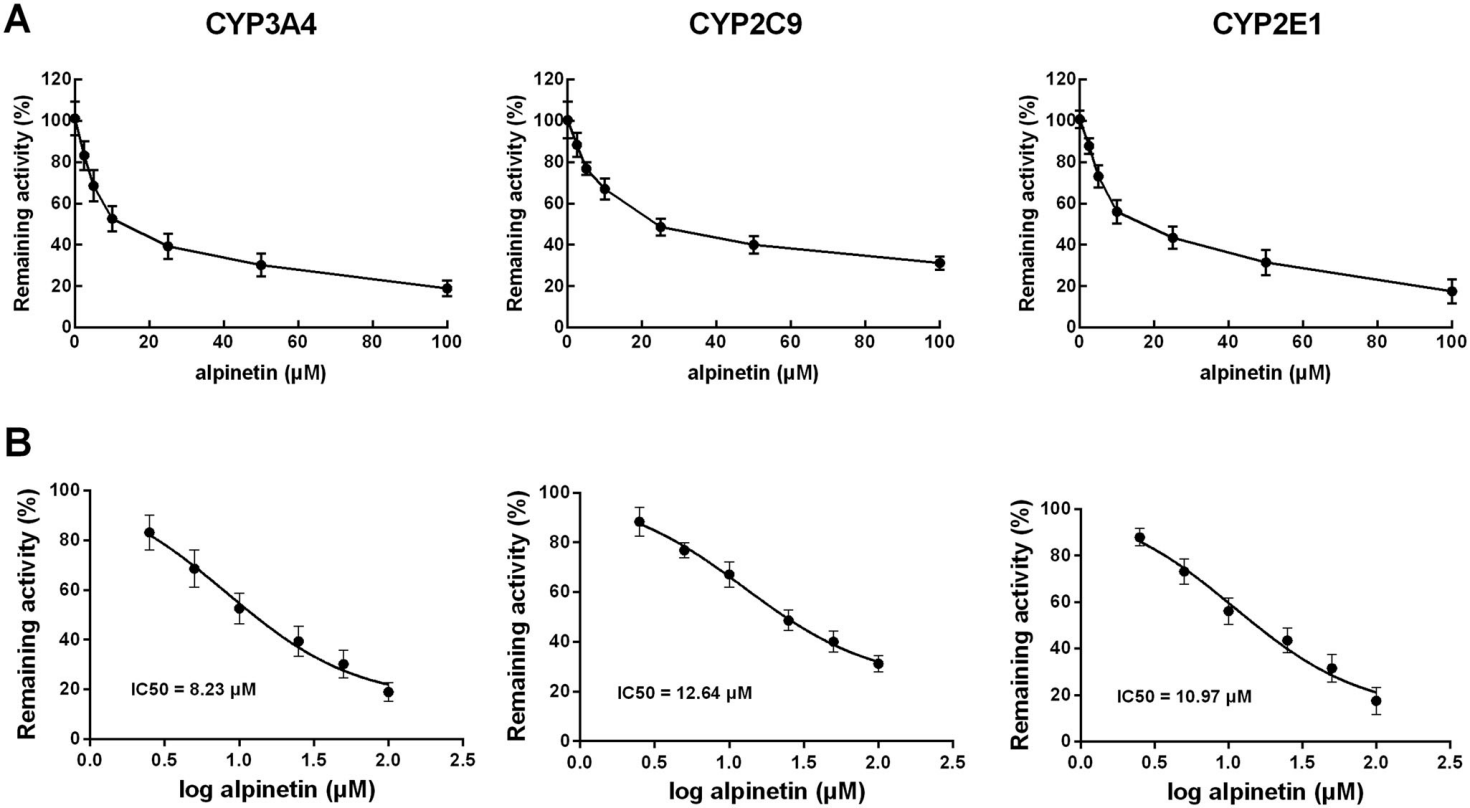
The dose-dependent inhibition of CYP3A4, 2C9, and 2E1. (A) The inhibition of CYP3A4, 2C9, and 2E1 was enhanced with the increasing concentration of alpinetin. (B) The IC_50_ values of CYP3A4, 2C9, and 2E1 were obtained as 8.23, 12.64, and 10.97 μM, respectively.

In the pooled liver microsomes, the activity of all CYP450 isoenzymes was obviously suppressed by their specific inhibitors (*p* < 0.01, Figure 2). While among the major isoenzymes, alpinetin showed a dramatically inhibitory effect on CYP3A4, 2C9, and 2E1 (*p* < 0.05, *p* < 0.01, Figure 2). Notably, the inhibition of these CYP450s was found to be concentration-dependent, which was enhanced with the increasing concentration of alpinetin (Figure 3(A)). The values of IC_50_ were obtained as 8.23 μM (CYP3A4) 12.64 μM (CYP2C9), and 10.97 μM (CYP2E1), respectively (Figure 3(B)).

### The inhibition model of CYP3A4

**Figure 4.**
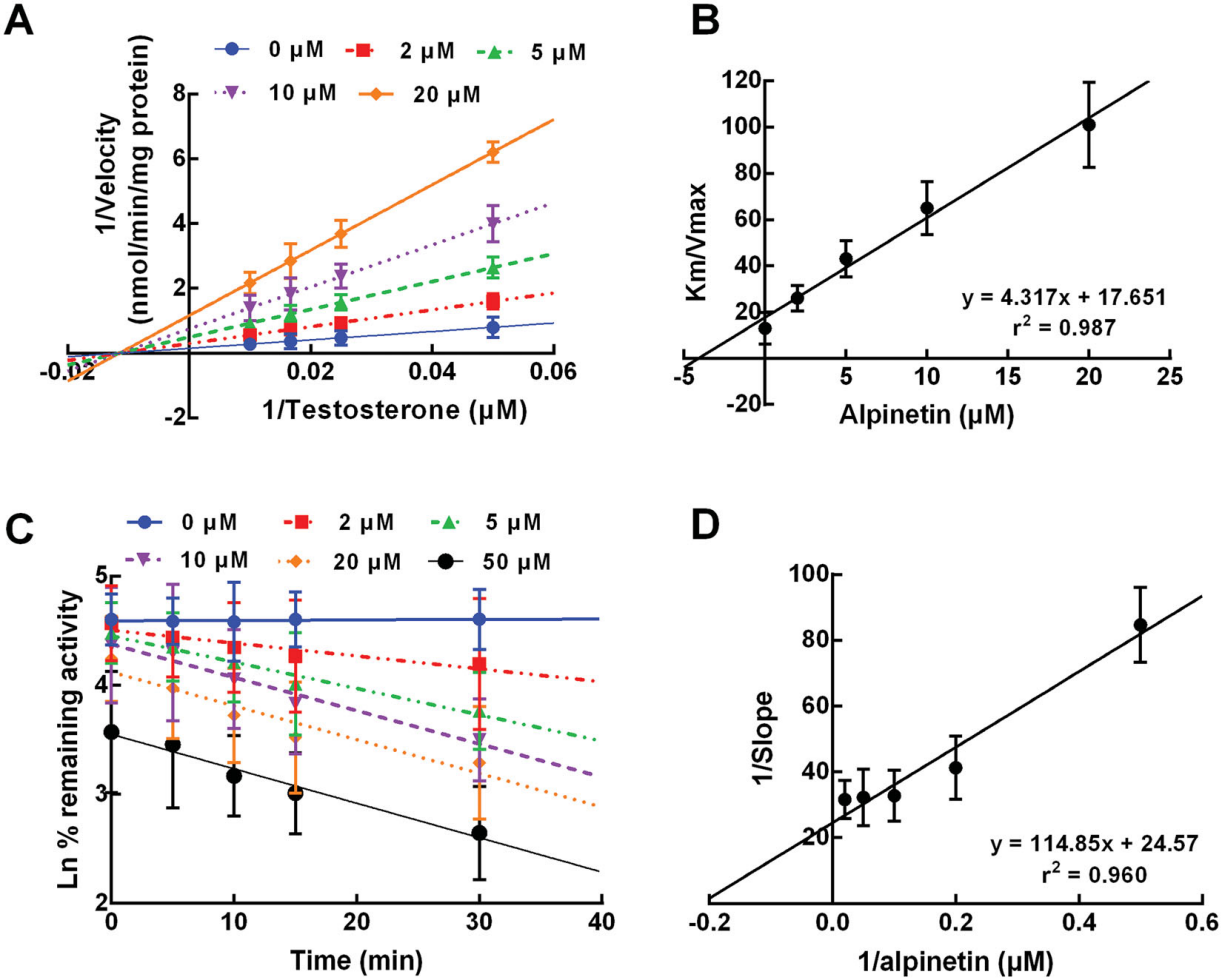
The inhibition model of CYP3A4. (A, B) The inhibition of CYP3A4 was non-competitive (A) with the *Ki* value of 4.09 μM (B). (C, D) The inhibition of CYP3A4 was time-dependent (C) with the *KI* and *Kinact* values of 4.67 min and 0.041 μM^−1^, respectively (D).

In the presence of 0, 2, 5, 10, and 20 μM alpinetin and various concentrations of testosterone, the inhibition of CYP3A4 was best fitted with the non-competitive model with a stable *Km*, where the velocity of CYP3A4 inhibition was found to be reduced with the increase of alpinetin concentration (Figure 4(A)). The *Ki* value of CYP3A4 inhibition by alpinetin was calculated as 4.09 μM (Figure 4(B)).

In addition, the inhibition of CYP3A4 by alpinetin also showed a time-dependent manner, which was enhanced by the elevating concentration of alpinetin (Figure 4(C)). The *KI* and *Kinact* value were obtained as 4.67 min and 0.041 μM^−1^, respectively (Figure 4(D)).

### The inhibition model of CYP2C9 and 2E1

**Figure 5.**
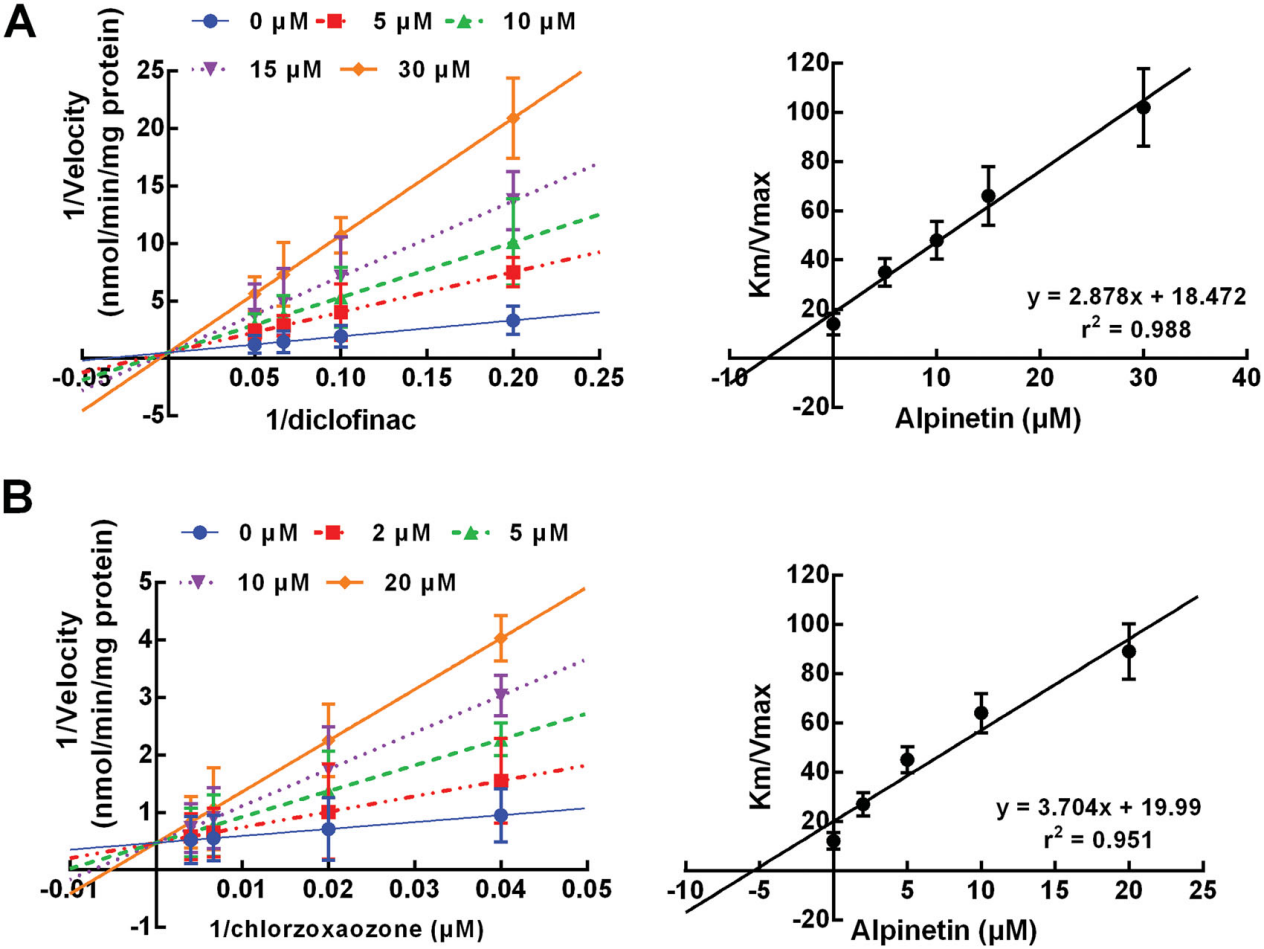
Both CYP2C9 (A) and 2E1 (B) were inhibited by alpinetine in a competitive manner with the *Ki* values of 6.42 and 5.40 μM, respectively.

The inhibition of CYP2C9 and 2E1 by alpinetin was observed to be displayed competitively with a stable *Vmax* (Figure 5(A)), with the *Ki* values of 6.42 and 5.40 μM, respectively (Figure 5(B)). Meanwhile, no time-dependent characteristics were observed during the inhibition of CYP2C9 and 2E1 (data not shown).

## Discussion

The alleviated effects of alpinetin on various human diseases have been reported in previous studies. The anti-inflammation effect of alpinetin has been illustrated in endometritis, bowel disease, and allergic asthma in mice (Liang et al. 2018; Wu, Li, et al. 2020; Yu et al. 2020), and its antitumor effect was observed in breast cancer and other malignant tumours (Wang et al. 2016; Zhao et al. 2018; Guo et al. 2020; Zhang, Guo, et al. 2020; Hou et al. 2021). With the widespread application of traditional Chinese herbs, the clinical treatment of breast cancer usually included herbal prescription, especially in the prevention and postoperative adjuvant therapies (Liu et al. 2019; Wang, Long, et al. 2020; Wang, Zhang, et al. 2020). The activity of CYP450s is a critical factor responsible for the therapeutic efficiency and is susceptible to external environmental conditions. Previously, several studies have unearthed the inhibitory or induced effect of various herbal extractions on the activity of different CYP450 isoenzymes. For example, lysionotin that is extracted from *Lysionotus pauciflorus* Maxim (Gesneriaceae) was revealed to inhibit the activity of CYP2C8, 2C19, and 3A4 in a dose-dependent manner (Li et al. 2020). *Terminalia chebula* Retz. dramatically suppressed the activity of CYP2E1 and 2C19, which induced its interaction with chlorzoxazone and omeprazole, which were metabolised by these two CYP450s (Wu, Dong, et al. 2020). As CYP450s could mediate the pharmacokinetic interactions between various herbs or drugs. Therefore, the effect of active extractions of herbs on CYP450 activity is of great significance for clinical prescriptions.

Herein, the inhibition of CYP3A4, 2C9, and 2E1 by alpinetin was observed, which was displayed in a concentration-dependent manner with various IC_50_ values. The inhibitory effect of alpinetin was demonstrated to be non-competitive in the present study, indicating that alpinetin did not affect the interaction between CYP3A4 and its substrates. Moreover, the inhibitory effect of alpinetin on CYP3A4 was enhanced by the incubation time. CYP3A4 is a vital member of the CYP450 family, which participates in the metabolism of numerous drugs, and was also involved in the adverse drug–drug interactions (Pal and Mitra 2006; Martinez-Jimenez et al. 2007). For instance, the inhibitory effect of Maha yogaraja guggulu and its major ingredients (*E*-guggulsterone and *Z*-guggulsterone) on CYP3A4 led to its interaction with conventional CYP3A4 substrates (Sabarathinam et al. 2021). Moreover, due the anti-breast cancer activity of alpinetin, its co-adminsitration with other antitumor drugs should draw special attention. According to reports, the latest small molecule targeted drug for breast cancer, palbociclib, a CDK4/6 inhibitor, its metabolism is closely related to CYP3A (Yu et al. 2017; Braal et al. 2021). Strong CYP3A inhibitor (itraconazole), inducer (Rifampicin), and sensitive CYP3A substrates (midazolam) have drug interactions with palbociclib, while moderate CYP3A inhibitors (diltiazem and verapamil) may increase blood palbociclib AUC by approximately 40% (Yu et al. 2017). Therefore, to avoid the risk of reduced effectiveness of palbociclib, the use of potent CYP3A4 inducers, including rifampicin, should be avoided. Similarly, if patients with certain diseases need to use CYP3A4 inhibitors including itraconazole and verapamil, the dose of palbociclib should be reasonably reduced. Hence, the interaction between alpinetin or its origin herbs and the drugs metabolised by CYP3A4 is of great potential to occur during their combination, which should draw special attention. Although CYP2C9 and 2E1 account for a small proportion in all CYP450 subtypes, they are also responsible for the metabolism of a huge number of drugs (Guengerich 2020; Wang, Gao, et al. 2020; Waring 2020). The observed inhibitory effects of alpinetin on CYP2C9 and 2E1 were found to be competitive, suggesting that alpinetin suppressed CYP2C9 and 2E1 activity via competing for the binding sites with substrates.

Previously, the role of genetic polymorphism in drug–drug interactions has been reported as a key factor that might affect the catalytic activity of CYP450 and therefore induce adverse drug–drug interaction (Bozina et al. 2009). The molecular mechanism underlying the effect of alpinetin on CYP450s activity needs further investigation. What’s more, the *in vivo* effect of alpinetin on CYP450s activity might be affected by the metabolites of alpinetin. A previous study has investigated the metabolites of alpinetin in rat plasma, urine, bile, and faeces, and identified a series of compounds with various functional groups, which was considered as the other potential mechanism underlying the inhibition of CYP3A4, 2C9, and 2E1 by alpinetin. On the other hand, the obtained *in vitro* findings directly evidenced the suppression of CYP3A4, 2C9, and 2E1 by alpinetin. However, the specific drug-drug interaction between alpinetin or its original source and their co-administrated drugs needs further *in vivo* validations.

Taken together, alpinetin dramatically inhibited CYP3A4, 2C9, and 2E1 *in vitro*. The administrated dose and incubation time were demonstrated to be two key factors that affected the degree of inhibition.
